# Sex-Related Disparities in Cardiac Masses: Clinical Features and Outcomes

**DOI:** 10.3390/jcm12082958

**Published:** 2023-04-19

**Authors:** Francesco Angeli, Luca Bergamaschi, Andrea Rinaldi, Pasquale Paolisso, Matteo Armillotta, Andrea Stefanizzi, Angelo Sansonetti, Sara Amicone, Andrea Impellizzeri, Francesca Bodega, Lisa Canton, Nicole Suma, Damiano Fedele, Davide Bertolini, Francesco Pio Tattilo, Daniele Cavallo, Ornella Di Iuorio, Khrystyna Ryabenko, Marcello Casuso Alvarez, Nazzareno Galiè, Alberto Foà, Carmine Pizzi

**Affiliations:** 1 Cardiology Unit, IRCCS Azienda Ospedaliera-Universitaria di Bologna, 40138 Bologna, Italy; 2 Department of Medical and Surgical Sciences (DIMEC), Alma Mater Studiorum, University of Bologna, 40138 Bologna, Italy; 3 Department of Advanced Biomedical Sciences, University Federico II, 80131 Naples, Italy

**Keywords:** cardio-oncology, cardiac masses, gender medicine, echocardiography

## Abstract

Background. Cardiac masses (CM) represent a heterogeneous clinical scenario, and sex-related differences of these patients remain to be established. Purpose: To evaluate sex-related disparities in CMs regarding clinical presentation and outcomes. Material and Methods. The study cohort included 321 consecutive patients with CM enrolled in our Centre between 2004 and 2022. A definitive diagnosis was achieved by histological examination or, in the case of cardiac thrombi, with radiological evidence of thrombus resolution after anticoagulant treatment. All-cause mortality at follow-up was evaluated. Multivariable regression analysis assessed the potential prognostic disparities between men and women. Results. Out of 321 patients with CM, 172 (54%) were female. Women were more frequently younger (*p* = 0.02) than men. Regarding CM histotypes, females were affected by benign masses more frequently (with cardiac myxoma above all), while metastatic tumours were more common in men (*p* < 0.001). At presentation, peripheral embolism occurred predominantly in women (*p* = 0.03). Echocardiographic features such as greater dimension, irregular margin, infiltration, sessile mass and immobility were far more common in men. Despite a better overall survival in women, no sex-related differences were observed in the prognosis of benign or malignant masses. In fact, in multivariate analyses, sex was not independently associated with all-cause death. Conversely, age, smoking habit, malignant tumours and peripheral embolism were independent predictors of mortality. Conclusions. In a large cohort of cardiac masses, a significant sex-related difference in histotype prevalence was found: Benign CMs affected female patients more frequently, while malignant tumours affected predominantly men. Despite better overall survival in women, sex did not influence prognosis in benign and malignant masses.

## 1. Introduction

Cardiac masses (CM) represent a complex clinical scenario, and data regarding their pathogenesis and epidemiology are lacking [1,2].

The term Cardiac Mass embraces four subgroups: benign tumours, primary malignant tumours, secondary malignant tumours and pseudotumours [3].

Primary cardiac tumours are a rare clinical condition, while metastatic tumours are frequently seen in oncological patients and are nearly twenty times more frequent than primary neoplasms [4]. Pseudo-tumours are also relatively common in everyday practice and include a wide range of clinical conditions (normal anatomic or congenital variants, bacterial or non-bacterial vegetations, inflammatory tumours and thrombi) [5,6,7].

Cardiac Masses have increased in incidence in recent years due to a more frequent application of second and third-level imaging techniques. They are now a frequent diagnostic dilemma for clinicians [8,9]. However, data regarding sex-related disparities in the epidemiological distribution or prognostic factors in patients with CMs are lacking [3].

Sex-related differences in incidence, clinical behaviour, and outcomes have been reported in the setting of various pathologies, such as cardiovascular and oncological diseases [10,11,12]. Several hypotheses have been proposed to explain these disparities.

Differences in genetic or molecular features between men and women could contribute to the different incidences of various cancers or in response to chemotherapy [13].

Moreover, sex hormones act as gene expression modulators and may therefore promote these variances in tumour phenotypes [11,13]. Oestrogens are also established protective factors for atherosclerosis and have several pleiotropic effects; this could explain the well-known differences in cardiovascular diseases such as coronary artery disease or cardiomyopathies between male and female patients [3,14,15].

Nevertheless, the relationship between sex and CMs is still unclear, and potential differences in incidence, clinical and imaging features between female and male patients remain to be investigated.

The aim of this study was to investigate the clinical, demographic and histotype differences between men and women with CMs and to assess sex-related outcomes at long-term follow-up.

## 2. Methods

### 2.1. Study Population

In this observational study, we included all consecutive patients who underwent non-invasive imaging investigations [trans-thoracic or trans-oesophageal cardiac ultrasound, Cardiac Computed Tomography (CCT) or Cardiac Magnetic Resonance (CMR)] for suspected CM at University Hospital Policlinico Sant’Orsola Malpighi, in Bologna, Italy, between January 2004 and November 2022.

Definitive diagnosis was achieved with histological specimens (resection or biopsy, either surgical or percutaneous) according to the American Society of Clinical Oncology/College of American Pathologists 2010 criteria or, in case of cardiac thrombi, by radiological evidence of thrombus resolution after appropriate anticoagulant treatment.

After the diagnostic workup, all patients were classified as primary (benign and malignant) and secondary (metastatic) tumours according to the 2015 World Health Organization (WHO) histological classification of tumours of the heart [2].

Pseudo-tumours, an extremely heterogenous group of clinical conditions, were defined as lesions not originating from a neoplastic transformation of a specific cell type [5]. Normal anatomic variants were excluded due to the impossibility of obtaining a histological diagnosis. 

In case of clinical suspicion of infective endocarditis, appropriate laboratory examinations and instrumental investigations were performed according to guidelines [16]. Only patients who did not meet the diagnostic criteria for infective endocarditis were included in our Registry.

All patients were treated in accordance with the Declaration of Helsinki and provided informed consent for the anonymous publication of scientific data. The study protocol was approved by the local Ethics Committee (Registration Number 102/2017/Oss/AOUBo).

### 2.2. Data Collection and Follow Up

Clinical and demographic data were collected for every patient, including age, sex, anthropometric data, cardiovascular risk factors and comorbidities. Each patient underwent an accurate diagnostic work-up, including clinical evaluation, laboratory testing and first [cardiac ultrasound], second [CCT, CMR] or third-level [18-Fluorodeoxyglucose Positron Emission Tomography (18-FDG-PET)] imaging techniques based on the specific clinical scenario. After the first global assessment, all patients were followed over time, and data were collected from clinical visits or telephone interviews.

### 2.3. Echocardiographic Features in Women vs. Men

For all patients, a 2D-echocardiogram, trans-thoracic or trans-oesophageal, when deemed appropriate, was performed according to the recommendations of the American Society of Echocardiography and the European Association of Cardiovascular Imaging [17,18]. The following parameters were assessed: location (left/right, atrium/ventricle, pericardium, great vessels), size, shape (regular/irregular), margins (well defined/irregular-if more than 50% of the border was clearly demarcated), mass characteristics (sessile-attached directly by the base and not raised upon a stalk, pedunculated-raised upon a stalk-or polylobate-having two or more lobes), mobility, infiltration [defined as disruption of surrounding tissue and extension of the mass across the pericardium into myocardium, with interruption of epicardial and endocardial contours or, alternatively, by the presence of at least one of the following echocardiographic features (i) evidence of a different echogenicity compared to the normal myocardium as infiltrating masses usually have a granular echocardiographic appearance; (ii) increased thickness in comparison with the adjacent myocardium; (iii) hypo/akinesia of a focal myocardial area compared to closest cardiac segments without coronary distribution suggestive of ischemic aetiology] [18] and pericardial effusion (defined as a fluid accumulation between the two pericardial layers) [19]. Left Ventricular Ejection Fraction (LVEF) was calculated using Simpson’s biplane method.

For the statistical analysis of echocardiographic parameters, we excluded patients with poor acoustic windows and masses that were not visible at the cardiac ultrasound. We evaluated potential disparities in the above-mentioned parameters between female and male patients.

### 2.4. Statistical Analysis

Continuous variables were presented as a median and inter-quartile range (IQR) or mean ± standard deviation (SD) according to values distribution evaluated using the Shapiro-Wilk test and the homogeneity of variance using Levene’s test. Categorical variables were summarized using absolute and relative frequencies.

We compared the clinical characteristics, laboratory parameters, and morphological features assessed at cardiac ultrasound in female and male patients with cardiac masses. Continuous variables were compared using Student’s t-test in case of a normal distribution or with the Mann-Whitney U test in case of abnormal distribution. A comparison of categorical variables was performed with the χ2 test. 

Outcome (all-cause death) was compared using the log-rank test and graphically using Kaplan-Meier curves between male and female patients with CM in the overall sample and divided by the nature of the CM (benign and malignant ones). Cox regression multivariate analysis was performed to determine independent predictors of all-cause death in the overall sample and considering covariates: age, sex, main cardiovascular risk factors, evidence of peripheral cardiac embolism and the nature of the CM (benign or malignant).

All the analyses were performed using Statistical Package for Social Sciences, version 25.0 (SPSS, PC version, Chicago, IL, USA). A 2-sided *p*-value < 0.05 was considered statistically significant.

## 3. Results

We enrolled patients from the Bologna Cardiac Masses Registry. Out of 379 CMs evaluated from January 2004 to November 2022, we excluded 40 cases without histological diagnosis and 18 with extracardiac masses confirmed at second or third-level imaging investigations, as shown in the study flow chart (Supplementary Figure S1). As a result, the final study population consisted of 321 patients, with 172 (54%) females and 149 (46%) males. The mean age was 59.8 ± 15.4 years (the range was 18–93 years).

Each patient completed the follow-up, with a median follow-up time of 20 months [IQR 8.00–75.00] and a maximum of 150 months (12.5 years).

### 3.1. Female vs. Male: Clinical Features

Baseline characteristics, cardiovascular risk factors and data regarding past medical history are reported in Table 1. Female patients were younger (*p* = 0.02), presented a lower Body Mass Index (BMI) (*p* = 0.01) and were less frequently smokers (*p* < 0.001). Other cardiovascular risk factors, comorbidities and past cardiovascular or oncological history were similar in the two groups. In the overall population, CMs were predominantly left-sided (54.2%) and observed at the atrial level (192 out of 321, 59.7%). The other neoformations were ventricular (13%), valvular (10.5%) or affected the pericardium (11.2%%) or great vessels (5.6%). These results were mainly driven by the high number of myxomas (114 out 321 masses, 35.5%) in our study population, which were predominantly left-sided (89.3%) and, more specifically, located in the left atrium (85.1%).

No differences between male and female patients were observed regarding mass localization or symptoms at clinical onset (Table 2). Interestingly, female patients were more prone to peripheral embolism (*p* = 0.003). Out of 43 peripheral embolism cases, 19 (44.2%) occurred in patients affected by cardiac myxoma.

Interestingly, women and men exhibited a similar cardioembolic risk determined by CHA2DS2-VASc scores, as non-sex-related risk factors were more represented in men.

Regarding the laboratory parameters, lower hemoglobin and creatinine levels and a higher Glomerular Filtration Rate were observed in female patients; no other differences were observed in the remaining laboratory results. 

### 3.2. Female vs. Males: Histological Subtypes

Out of 321 patients, 99 (30.8%) were affected by malignant tumours, either primary or metastatic, which were more common in the male population (43% vs. 20.3%, *p* < 0.001). On the contrary, benign tumours were less frequently diagnosed in men compared to women (32.3% vs. 58.7%, *p* < 0.001). No differences were observed in pseudotumours’ distribution between males and females (24.8% vs. 20.1, *p* = 0.43).

Despite being more represented in women (82 out of 114 cases, with a female-to-male ratio of 2.6:1), the most frequent tumour was myxoma in both men and women (32 out of 149 masses, 21.5%, and 82 out of 172, 47.7%, respectively).

The distribution of histological subtypes in female and male population is described in Table 3. 

Thirty-five patients were enrolled throughout the radiological confirmation of thrombus resolution after appropriate anticoagulant treatment, while in 2 cases the diagnosis of thrombotic neoformation was obtained after surgery.

### 3.3. Female vs. Male: Echocardiographic Features

For the analysis of echocardiographic features of CMs we excluded 35 patients with poor acoustic windows or with masses not visualizable at cardiac ultrasound. A complete ultrasound assessment of CM was therefore performed in 286 patients. In 92 patients (32.2%) a trans-oesophageal echocardiography was performed.

Echocardiographic features were significantly different between male and female patients (Table 4). In men, CMs showed greater dimension and were more frequently sessile with irregular margins, immobile and infiltrated in comparison to cardiac neoformations in female patients. Additionally, male patients presented more frequently with pericardial effusion (*p* = 0.001). Finally, women presented a slightly higher LVEF than men (*p* = 0.001).

Out of 114 myxomas, 111 (97.3%) were visualized by cardiac ultrasound. Regarding morphological features, no differences were observed between female and male patients with myxoma (Supplementary Table S1). 

### 3.4. Outcomes according to Sex in Cardiac Masses

After a median follow-up of 20 months [IQR 8.00–75.00], the overall all-cause mortality was 28.1% (90 deaths out of 321).

In the overall study population the Kaplan-Meier curve analysis women showed a significantly greater survival during follow-up in comparison to men (Log-rank test = 7.242, *p* = 0.007) (Supplementary Figure S2).

**Figure 1:**
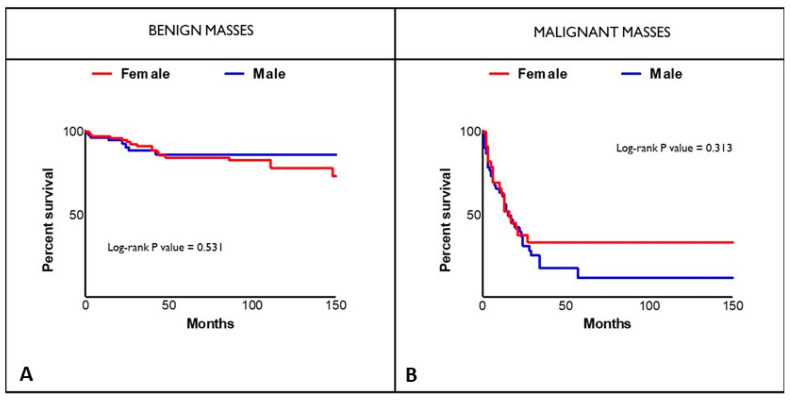
Kaplan-Meier Survival Curves. (**A**) Survival in patients with benign neoformations according to gender. (**B**) Survival in patients with malignant tumours according to gender.

Nevertheless, we did not observe significant differences in the survival of benign and malignant lesions and in the specific histological subgroups between male and female patients (Figure 1 and Table 5, respectively).

In fact, at multivariate Cox regression analysis corrected for age, sex, smoking habit, hypertension, dyslipidemia, diabetes mellitus, peripheral artery disease, malignant tumour (primary or metastatic) and peripheral embolism at clinical onset, sex did not emerge as an independent predictor of mortality (Table 6). Independent predictors of death during follow up are reported in Table 6.

## 4. Discussion

The aim of this study was to describe the population of Cardiac Masses of the Bologna Registry and evaluate potential sex-related differences in such a complex clinical scenario.

In summary, our main findings are (a) female patients were younger and affected more frequently by benign cardiac masses with myxoma as the most common histotype, (b) metastatic tumours were more commonly diagnosed in men, (c) female patients with cardiac masses had a better overall survival compared to men during follow-up, but this survival benefit was not significant considering malignant or benign subgroups. In fact, sex was not an independent predictor of survival in patients with cardiac masses. (d) However, clinical features associated with poor prognoses, such as peripheral embolism, were observed in women more often while some echocardiographic parameters suggestive of malignant tumours [19,20] were more prevalent in our male population.

### 4.1. Sex-Related Differences in Clinical Manifestations

Cardiac masses represent a rare clinical condition, and little is known regarding their pathogenesis, and epidemiological and prognostic factors. Assessing potential clinical differences could help clinicians during the diagnostic work-up of this complex scenario, which is often a time-dependent disease [21,22]. Sex differences have been observed in other cardiovascular diseases, such as coronary artery disease, valvulopathies or arrhythmias. However, sex-related disparities in cardiac masses have still not been clearly established [12,14,15].

In our study population, benign tumours were the most frequent lesions in both male and female patients, with cardiac myxoma being the most prevalent tumour. However, this histotype was more represented in women (82 out of 114 cases, with a female-to-male ratio of 2.6:1).

Our results are consistent with previously published studies, which reported a female-to-male ratio varying from 2:1 to 3:1 for this tumour [23], though little is known about the role of sex in the pathogenesis of cardiac myxoma. A possible explanation could be the role of oestrogens in modulating genetic expression in the neoplastic process [24]. 

Nevertheless, in our study, male patients were older and affected more frequently by metastatic tumours. These results were mainly driven by a higher frequency of systemic malignant cancer in men, especially in the elderly [10].

Despite no significant difference in the location of masses between men and women, peripheral embolism was more frequently observed in female patients. This represents a potentially deadly complication of both benign and malignant neoformations that worsens the patient’s prognosis and quality of life. The higher prevalence of myxoma in women could be one of the possible explanations for the greater rate of embolism observed in this population, as it is a tumour with friable tissue and, therefore, more prone to embolism [25].

### 4.2. Sex-Related Differences in Clinical Outcomes

Outcomes in patients with CMs vary on the basis of masses’ nature, embolic complications and, in the case of malignant tumours, the cardiac extension and the presence of systemic metastatic tumours [3,21,26,27].

Patients with malignant tumours have a poor prognosis with a median survival ranging from 6 to 18 months, as reported in prior studies [27]. In our population, we observed 90 deaths during follow-up, mainly in patients with malignant tumours (63 out of 90 deaths, 70%).

In the overall population, female patients showed a better survival rate at follow-up compared to males, but the prognosis of both benign and malignant neoformations did not differ between males and females. The overall worse prognosis in men with CMs could therefore be explained by the higher incidence of malignant tumours in this population and not by sex itself. As a matter of fact, sex did not remain an independent predictor of death in multivariate analyses in our cohort.

**Figure 2:**
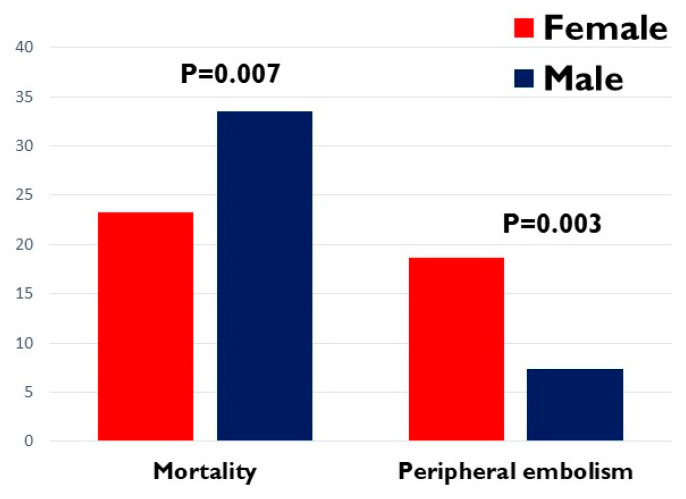
Mortality and peripheral embolism according to gender. Histograms demonstrating the different mortality and peripheral embolism incidence in female and male patients.

However, peripheral embolism occurred more frequently in women and in the multivariate analysis, it emerged as an independent predictor of death. Therefore, it should be interpreted as a red flag even in the case of benign mass and could represent a useful marker to discriminate patients at higher risk in a population with an overall better prognosis (Figure 2).

### 4.3. Sex-Related Differences in Echocardiographic Features

Cardiac ultrasound represents the first line imaging technique in the diagnostic work-up of cardiac masses [28]. In fact, it offers valuable information regarding size, shape, morphological features and location of the mass, as well as its relationship with surrounding structures [19,20]. Furthermore, trans-oesophageal echocardiography provides a higher diagnostic accuracy, as it offers a more detailed mass assessment and characterizes better lesions located in the atria [18,19].

Cardiac ultrasound is crucial in the identification of patients who may benefit from second (Cardiac Computed Tomography and Cardiac Magnetic Resonance) and third-level (18- Fluorodeoxyglucose Positron Emission Tomography) imaging techniques [20].

In contrast, echocardiography offers poor tissue characterization and is often unable to define the masses’ nature; nevertheless, some morphological features associated with malignant tumours have been previously described and included in echocardiographic scores to improve its diagnostic accuracy, therefore avoiding potential unnecessary costs and radiation exposure of second and third level imaging techniques [19,20].

In our population, CMs in male patients more frequently presented infiltration to the surrounding tissues, irregular margins, and greater dimensions compared to masses in female patients. Moreover, pericardial effusion was more often seen in the male population. These hallmarks are reported to be associated with malignant lesions and are therefore more prevalent in male patients due to the higher prevalence of cancer, either primary or metastatic, in this population [19,20,29].

## 5. Study Limitations

This study has some limitations that deserve to be considered. First, our study is a single-centre experience with a relatively limited sample size, despite representing the largest series with CMs with histological diagnosis.

Second, in our population, the prevalence of malignant tumours may be underestimated because in advanced neoplasms, further diagnostic investigations are not often performed, and histopathological diagnosis is therefore not obtained.

Finally, surgical procedures and diagnostic techniques have improved in the last 18 years of patient recruitment; it is possible that the diagnostic accuracy of imaging techniques and survival rates have changed in these years.

## 6. Conclusions

This study on a large group of cardiac masses with histological diagnosis demonstrates the important role of sex in this clinical scenario. Women with CMs were younger and predominantly affected by benign neoformations, while malignant tumours affected mostly men.

Despite a better overall prognosis, the female sex did not influence the prognosis of benign or malignant neoformations. 

However, some clinical red flags of poor outcomes, such as peripheral embolism, occurred more frequently in women. This may be useful to discriminate patients at higher risk despite an overall better prognosis driven by the cardiac masses’ histotype.

## Figures and Tables

**Table 1:** Comparison of demographic and anamnestic features between female and male patients affected by cardiac masses.

	Total Population N = 321	Female Patients N = 172	Male Patients N = 149	*p*-Value
Age in years, mean ± SD	59.8 ± 15.4	61.4 ±15.7	57.9 ± 15.4	0.02
BMI in kg/m^2^, mean ± SD	25.4 ± 4.4	24.8 ± 4.1	26.3 ± 3.8	0.01
Cardiovascular risk factors				
Smoking habit, n (%)	160 (49.8)	60 (34.8)	100 (66.9)	<0.001
Hypertension, n (%)	180 (56.1)	97 (56.4)	83 (55.7)	0.84
Dyslipidemia, n (%)	140 (43.6)	81 (47)	59 (39.9)	0.22
DM, n (%)	48 (14.9)	24 (13.9)	24 (16.2)	0.27
Medical History				
Congestive Heart failure, n (%)	40 (12.5)	15 (8.7)	25(16.8)	0.06
Prior stroke, n (%)	70 (21.8)	32 (18.6)	28 (18.8)	0.12
Vasculopathy, n (%)	78 (24.8)	42 (24.4)	36 (24.2)	0.19
History of neoplasia, n (%)				0.53
None	210 (65.4)	116 (67.4)	94 (63.1)	
Benign	32 (9.9)	21 (12.2)	11 (7.4)	
Malignant	79 (24.7)	41 (23.8)	38 (25.5)	
CHA2DS2-VASc, mean ± SD	2.6 ± 1.7	2.7 ± 1.6	2.4 ± 1.7	0.06

Continuous variables are presented as mean ± Standard Deviation (SD); categorical ones as n (%). Abbreviations: BMI: body mass index; DM: Diabetes Mellitus.

**Table 2:** Comparison of clinical features, laboratory results and mass location between female and male patients with cardiac neoformations.

	Total Population N = 321	Female Patients N = 172	Male Patients N = 149	*p*-Value
Clinical features				
Malignant lesion, n (%)	99 (30.8)	35 (20.3)	64 (43)	<0.001
Accidental diagnosis, n (%)	137 (42.7)	71 (41.3)	66 (44.3)	0.56
Dyspnea, n (%)	129 (40.2)	73 (42.4)	56 (37.6)	0.36
NYHA Classification, n (%)				0.26
Class I–II	244 (76)	132 (76.8)	112 (75.2)	
Class III–IV	77 (24)	40 (23.2)	37 (24.8)	
Chest pain, n (%)	52 (16.2)	30 (17.4)	22 (14.8)	0.49
Peripheral embolism, n (%)	43 (13.4)	32 (18.6)	11 (7.4)	0.003
Pulmonary embolism, n (%)	44 (13.7)	24 (13.9)	20 (13.4)	0.1
Location, n (%)				0.087
Right cardiac chambers, n (%)	93 (29)	45 (26.2)	48 (32.2)	
Left cardiac chambers, n (%)	174 (54.2)	104 (60.5)	70 (47)	
Pericardium, n (%)	36 (11.2)	15 (8.7)	21 (14)	
Great Vessels, n (%)	18 (5.6)	8 (4.6)	10 (6.8)	
Laboratory parameters				
Creatinine in mg/dL, mean ± SD	1.04 ± 0.67	0.88 ± 0.65	1.22 ±0.8	0.002
GFR in mL/min, mean ± SD	80.4 ±26.3	84.3 ± 23.1	75.8 ±28.9	0.003
Hb in g/dL, mean ± SD	12.7 ± 2	12.4 ± 1.6	13.2 ± 2.1	0.001
WBC in n/mmc, mean ± SD	8522 ± 3846	8472 ±3121	8579 ±4114	0.36
LDH in U/L, median [IQR]	231 [155–289]	222 [143–272]	244 [135–279]	0.67
CRP in mg/dL, median [IQR]	1.89 [3.5–0.45]	1.5 [3.2–0.3]	1.7 [3.4–0.7]	0.07

Continuous variables are presented as mean ± Standard Deviation (SD) or median with inter-quartile range (IQR) when appropriate; categorical ones as n (%). Abbreviations: NYHA: New York Heart Association; GFR: glomerular filtration rate; Hb: hemoglobin; WBC: white blood cells; CRP: C-reactive protein; LDH: lactate dehydrogenase.

**Table 3:** Histological characterization of cardiac neoformations according to female and male patients.

	Total Population N = 321	Female Patients N = 172	Male Patients N = 149	*p*-Value
Benign tumours	149 (46.4)	101 (58.7)	48 (32.2)	<0.001
Myxoma	114 (76.5)	82 (81.2)	32 (64.6)	
Fibroelastoma	23 (15.4)	12 (11.2)	11 (22.9)	
Lipoma	5 (3.2)	2 (2)	3 (6.3)	
Fibroma	3 (2)	2 (2)	1 (2)	
Paraganglioma	3 (2)	2 (2)	1 (2)	
Hamartoma	1 (0.7)	1 (0.9)	0 (0)	
Pseudotumours	73 (22.7)	36 (20.1)	37 (24.8)	0.43
Thrombus	37 (50.7)	20 (55.56)	17 (45.9)	
Cyst	10 (13.7)	5 (13.9)	5 (13.5)	
Valvular nodule	10 (13.7)	3 (8.3)	7 (18.9)	
Lipomatosis	6 (8.3)	4 (11.1)	2 (5.4)	
Reactive inflammatory process	6 (8.3)	3 (8.3)	3 (8.1)	
Calcification	3 (4.1)	0 (0)	3 (8.1)	
Cystic atrioventricular node tumour	1 (1.7)	1 (2.8)	0 (0)	
Primary malignant tumours	35 (10.9)	15 (8.7)	20 (13.4)	0.21
Sarcoma	31 (88.6)	14 (93.3)	17 (85)	
Lymphoma	3 (8.6)	0 (0)	3 (15)	
Mesothelioma	1 (2.8)	1 (6.7)	0 (0)	
Metastatic tumours	64 (19.9)	20 (11.6)	44 (29.5)	<0.001
Lymphoma	23 (35.9)	5 (25)	18 (40.9)	
Lung carcinoma	8 (12.5)	2 (10)	6 (13.6)	
Sarcoma	7 (10.9)	2 (10)	5 (11.4)	
Renal and urological tumours	7 (10.9)	3 (15)	4 (9.1)	
Melanoma	6 (9.3)	2 (10)	4 (9.1)	
Hepatocellular carcinoma	5 (7.8)	1 (5)	4 (9.1)	
Colon carcinoma	4 (6.3)	1 (5)	3 (6.8)	
Gynaecological tumours	3 (4.7)	3 (15)	0	
Plasmacytoma	1 (1.6)	1 (5)	0	

**Table 4:** Comparison of echocardiographic features between male and female patients affected by cardiac neoformations.

	Total Population N = 286	Female Patients N = 155	Male Patients N = 131	*p*-Value
Ventricular parameters				
Ejection Fraction in %, mean ± SD	60.4 ± 9.7	62.3 ±8.3	58.4 ± 11.2	0.001
LV EDD in mm, mean ± SD	46.7 ±6.4	45 ± 5.7	48.7 ± 6.6	<0.001
LV EDV in mm^3^, mean ± SD	92.9 ± 35.1	81 ± 24.8	107.2 ± 40.9	<0.001
LAV in mL, mean ± SD	51 ± 5	48 ± 3	55 ± 4	0.067
DDF, n (%)	13 (4.5)	7 (4.6)	6 (4.5)	0.98
sysPAP in mmHg, mean ± SD	34.9 ± 15	35.8 ± 18	32.8 ± 12.3	0.79
Mitral regurgitation, n (%)				0.87
Mild	160 (55.9)	91 (58.7)	69 (52.7)	
Moderate/severe	40 (14)	20 (12.9)	20 (15.3)	
Mitral stenosis, n (%)				0.2
Mild	8 (2.8)	6 (3.9)	2 (1.5)	
Moderate/severe	12 (4.2)	8 (5.2)	4 (3.1)	
Aortic regurgitation, n (%)				0.56
Mild	61 (21.3)	36 (23.2)	25 (19.1)	
Moderate/severe	13 (4.5)	8 (5.2)	5 (3.8)	
Aortic stenosis, n (%)				0.6
Mild	22 (7.7)	11 (7.1)	11 (8.4)	
Moderate/severe	5 (1.7)	4 (2.6)	1 (0.8)	
Tricuspid regurgitation, n (%)				0.54
Mild	159 (55.6)	88 (56.8)	71 (54.2)	
Moderate/severe	26 (9.1)	10 (7.6)	16 (10.3)	
Pericardial effusion, n (%)	63 (22)	21 (13.5)	42 (32.1)	0.001
Mild, n (%)	34 (53.9)	15 (71.4)	19 (45.2)	
Moderate/severe, n (%)	29 (46.1)	6 (28.6)	23 (54.7)	
Mass features				
Infiltration, n (%)	57 (19.9)	19 (12.3)	38 (29)	0.001
Max CM diameter in mm, mean ± SD	36 ±19.6	33.8 ±15.9	38.6 ±22.9	0.02
Inhomogeneity, n (%)	73 (25.5)	35 (22.6)	38 (29)	0.22
Irregular margins, n (%)	72 (25.2)	24 (15.5)	48 (36.6)	<0.001
Mobility, n (%)	143 (50)	88 (56.8)	55 (42)	0.04
Sessile mass, n (%)	129 (45.1)	62 (40)	67 (51.1)	0.02
Polylobate mass, n (%)	72 (25.2)	29 (18.7)	43 (32.8)	0.003

Continuous variables are presented as mean ± Standard Deviation (SD); categorical ones as n (%). Abbreviations: LVEDD: Left Ventricular End-Diastolic diameter; LVEDV: Left Ventricular End-Diastolic Volume; LAV: Left Atrial Volume; DDF: diastolic dysfunction; sPAP: systolic pulmonary artery pressure.

**Table 5:** Mortality during follow up among histology subgroups according to sex.

	Total Population	Female Patients	Male Patients	*p*-Value
Benign tumours	17/149 (11.4)	14/101 (13.9)	3/48 (6.3)	0.27
Primary Malignant tumours	23/35 (65.7)	8/15 (53.3)	15/20 (75.5)	0.12
Pseudotumours	10/73 (13.7)	5/36 (13.9)	5/37 (13.5)	0.62
Metastatic tumours	40/64 (63)	13/20 (65)	27/44 (61.4)	0.51

**Table 6:** Univariate and multivariable logistic regression model showing the clinical predictors of death during follow up in patients with Cardiac Masses.

	Univariate Analysis				Multivariate Analysis			
	Odds Ratio	Standard Error	95% CI	*p* Value	Odds Ratio	Standard Error	95% CI	*p* Value
Age	1.02	0.008	1.04–1.034	0.01	1.03	0.08	1.04–1.01	<0.001
Male Sex	1.76	0.21	2.66–1.157	0.008	-	-	-	-
Smoking	1.38	0.21	2.09–0.91	0.132	1.55	0.22	2.35–1.001	0.049
Hypertension	0.81	0.22	1.24–0.5	0.33	-	-	-	-
Dyslipidaemia	1.11	0.21	1.69–0.73	0.62	-	-	-	-
Diabetes Mellitus	0.75	0.28	1.29–0.44	0.3	-	-	-	-
Peripheral artery disease	1.22	0.24	1.95–0.76	0.41	-	-	-	-
Malignant tumour	9.98	0.24	15.9–6.26	<0.001	13.32	0.26	21.98–8.07	<0.001
Peripheral embolism	0.97	0.3	1.74–0.54	0.914	1.94	0.32	3.61–1.05	0.035

CI: Confidence Interval.

## Data Availability

The datasets used and/or analyzed during the current study are available from the corresponding author on reasonable request.

## Supplementary Materials

The following supporting information can be downloaded at: https://www.mdpi.com/article/10.3390/jcm12082958/s1, Figure S1: Study flow chart; Figure S2: Kaplan-Meier Survival Curves in the overall population according to gender. In the lower panel, the table shows the number of patients at risk during follow up; Table S1: Comparison of echocardiographic features between male and female patients affected by cardiac myxoma.

## Abbreviations

BMI	Body Mass Index
CCT	Cardiac Computed Tomography
CM	Cardiac Masses
CMR	Cardiac Magnetic Resonance
LVEF	Left Ventricular Ejection Fraction
WHO	World Health Organization
18-FDG-PET	18-Fluorodeoxyglucose Positron Emission Tomography
