# Supplementary material for: Sex-Related Disparities in Cardiac Masses: Clinical Features and Outcomes
Source: J Clin Med. 2023 Apr 19;12(8):2958. doi: 10.3390/jcm12082958 (PMC10142943; doi:10.3390/jcm12082958)
Supplement: Supplementary file 1 [file jcm-12-02958-s001.zip › jcm-2244565-supplementary.pdf]

## SUPPLEMENTARY MATERIALS

## SUPPLEMENTARY FIGURE LEGEND

**Supplementary Figure S1.** Study flow chart.

**Supplementary Figure S2.** Kaplan-Meier Survival Curves in the overall population according to gender. In the lower panel, the table shows the number of patients at risk during follow up.

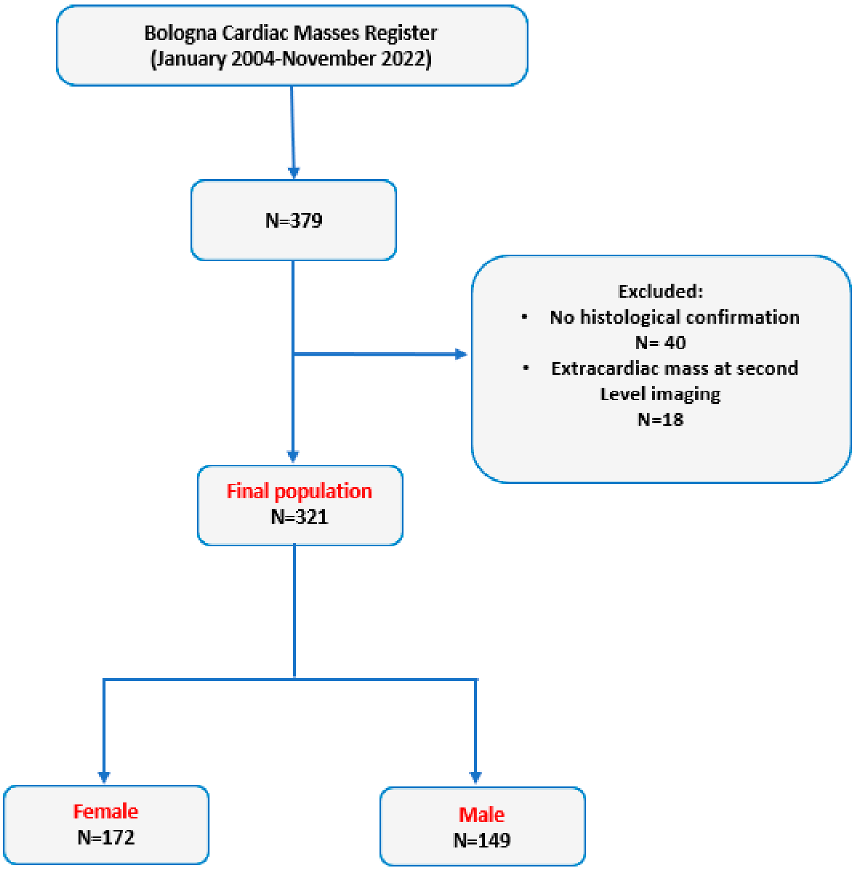


**Supplementary Figure S1.** Study flow chart.

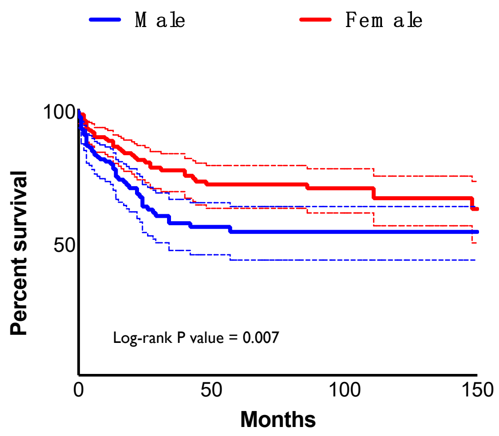


| Time | 0 | 50 | 100 | 150 |
|---|---|---|---|---|
| | 149 | 36 | 18 | 10 |
| | 172 | 65 | 44 | 16 |

**Supplementary Figure S2. Kaplan-Meier Survival Curves** in the overall population according to gender. In the lower panel, the table shows the number of patients at risk during follow up.

## SUPPLEMENTARY TABLES

**Supplementary Table S1**. Comparison of echocardiographic features between male and female patients affected by cardiac myxoma.

| | **Total population N = 111** | **Female patients N = 80** | **Male patients N = 31** | **P-value** |
|---|---|---|---|---|
| **Mass features** | | | | |
| Infiltration, n (%) | 0 (0.0) | 0 (0.0) | 0 (0.0) | - |
| Max CM diameter in mm, mean ±SD | 34.8 ±1.3 | 33.8 ±1.5 | 34.6 ±2.4 | 0.5 |
| Inhomogeneity, n (%) | 18 (16.2) | 7 (8.8) | 11 (35.4) | 0.22 |
| Irregular margins, n (%) | 13 (11.7) | 7 (8.8) | 6 (19.4) | 0.12 |
| Mobility, n (%) | 91 (82) | 64 (80) | 27 (87.1) | 0.38 |
| Sessile mass, n (%) | 29 (26.1) | 22 (27.5) | 7 (22.6) | 0.59 |
| Polylobate mass, n (%) | 14 (12.6) | 9 (11.3) | 5 (16.1) | 0.49 |

Continuous variables are presented as mean ± Standard Deviation (SD); categorical ones as n (%). Abbreviations: CM: Cardiac Mass.
